# Validation of Repeated Endothelial Function Measurements Using EndoPAT in Stroke

**DOI:** 10.3389/fneur.2017.00178

**Published:** 2017-05-03

**Authors:** Aina S. Hansen, Jawad H. Butt, Sonja Holm-Yildiz, William Karlsson, Christina Kruuse

**Affiliations:** ^1^Department of Neurology, Stroke Unit, Neurovascular Research Unit, Herlev-Gentofte University Hospital, Herlev, Denmark

**Keywords:** endothelial function, stroke, validation, EndoPAT, vasomotor reactivity

## Abstract

**Background:**

Decreased endothelial function (EF) may be a prognostic marker for stroke. Measuring pharmacological effects on EF may be of interest in the development of personalized medicine for stroke prevention. In this study, we assessed the reliability of repeated EF measurements using a pulse amplitude tonometry technology in acute stroke patients. Similarly, reliability was tested in healthy subjects devoid of vascular disease to estimate reactivity and reliability in a younger non-stroke population.

**Materials and methods:**

EF was assessed using the EndoPAT2000 in 20 healthy volunteers (men 50%, mean age 35.85 ± 3.47 years) and 21 stroke patients (men 52%, mean age 66.38 ± 2.85 years, and mean NIHSS 4.09 ± 0.53) under standardized conditions. EF was measured as the reactive hyperemia index (RHI), logarithm of RHI (lnRHI), and Framingham RHI (fRHI). Measurements were separated by 1.5 and 24 h to assess same-day and day-to-day reliability, respectively.

**Results:**

Fair to moderate correlations of measurements [intraclass correlation coefficient (ICC)_same-day_ 0.29 and ICC_day-to-day_ 0.52] were detected in healthy subjects. In stroke patients, we found moderate to substantial correlation of both same-day and day-to-day repeated measurements (ICC_same-day_ 0.40 and ICC_day-to-day_ 0.62). fRHI compared with RHI and lnRHI showed best reliability.

**Conclusion:**

Repeated measurements of fRHI in stroke patients show moderate reliability on same-day and substantial on day-to-day measurements. Likewise, in healthy subjects there was substantial reliability on day-to-day measurement, but only moderate on same-day measurements. In general, day-to-day correlation of repeated EF measurements was far better than that of same-day measurements, which ranged from poor to moderate depending on the specific outcome measure of EF. A possible carryover effect should be considered if same-day repeated testing of drug effects is applied in future studies.

## Background

Studying drug effects on endothelial function (EF) in stroke patients could add to the understanding of stroke pathophysiology, effects of new treatment, and identify patients at increased risk of stroke.

Reduced EF is associated with an increased risk of stroke (1–3), cardiovascular diseases (4), hypertension (5), hypercholesterolemia (5), type II diabetes (6, 7), and obesity (8). However, methods for evaluating EF are not yet agreed upon (9–12).

Endothelial function determination in large-scale risk stratification of stroke patients must be reliable, non-invasive, and widely applicable, as well as a good predictor for the risk of future vascular events. In addition, improvement in test results must be related to a subsequent reduction in risk of stroke. If fulfilling these requirements, measurement of EF could be a new tool for further evaluating and advising stroke patients.

Non-invasive measurements of EF have been used extensively in vascular research (9). The most frequently applied method is flow-mediated dilation (FMD) assessed by brachial artery ultrasound (13). This method is reliable on repeated measurements (14) and associated with cardiovascular risk factors (5, 15, 16), but show high dependency on operator experience level with special training required (12). Computerized analysis of pulse amplitude tonometry (PAT) in the index finger after reactive hyperemia has gained increasing interest as an easy accessible non-invasive method for assessment of EF (17, 18). Such automatized method for measuring EF requires little training of the operator and is thus easy to apply in studies without extensive training of personal.

Digital vascular response to reactive hyperemia is found to be predominantly dependent on nitric oxide-mediated vasodilation in healthy subjects (19) and has been shown to be attenuated in patients with coronary microvascular endothelial dysfunction (20). Large community-based cohort studies reported an association between EF and cardiovascular risk factors (5, 21). However, the risk factor profile for FMD and digital vascular response differed, with the latter more associated with metabolic dysfunction. Hence, FMD and EndoPAT probably reflect different aspects of vascular function in selected vascular beds and vessel size (5, 21). A study investigating the relationship of digital EF and stroke subtypes according to the TOAST classification (22) found variations in EF according to stroke subtypes with best EF in patients with cardioembolic stroke and worst in lacunar and large artery stroke (23).

Previous studies found good correlation of repeated digital EF measurements using PAT technology performed on different days in healthy volunteers (24–28) and in patients with coronary artery disease (CAD) (14). However, the technique has not yet been validated in stroke patients. Validation in stroke patients is important due to vascular instability in the acute phase poststroke that may affect the reliability of measurements (29, 30).

The primary outcome of this study was to determine the reliability of repeated digital EF measurements in patients with acute stroke on same-day and day-to-day measurements. In addition, we wish to assess repeated digital EF measurements in healthy volunteers to correlate our results with those previously published.

The results from this study are important for calculating sufficient sample sizes for future studies using this technique in stroke patients and to assess if repeated PAT measurements are valid in acute stroke.

## Materials and Methods

### Subjects

Only subjects above 18 years of age were included after signing informed consent. Twenty healthy subjects were recruited from the hospital staff and the local community. They did not take medications and had no prior history of cardiovascular disease, arrhythmias, diabetes, hypercholesterolemia, or hypertension. Twenty-two patients with non-cardioembolic acute stroke were recruited from patients admitted to the Stroke Unit, Department of Neurology, Herlev-Gentofte University Hospital. Stroke was defined as acute neurological deficits lasting for more than 24 h with radiological evidence of a corresponding acute ischemic brain lesion (CT or MRI), and stroke severity was assessed by NIHSS. Measurements were concluded within 5 days of stroke onset. Five of 22 stroke patients used one or more vasoactive medication (calcium antagonists: 3; angiotensin-receptor blockers: 3). On the days of examination, administration of medication was postponed until measurements were concluded.

### Procedure

Subjects were instructed to fast for 8 h, and further to refrain from smoking, alcohol- and xanthine-containing food, and beverages for 12 h prior to EF testing. Measurement of EF was performed two times on one day, separated by 1.5 h, and on the following day, separated by exactly 24 h.

Endothelial function and arterial stiffness were assessed by digital plethysmography using the EndoPAT2000-device (Itamar Medical Ltd., Caesarea, Israel). Measurements were conducted in accordance with conditions specified by the manufacturer. All tests were performed in the supine position in quiet surroundings, on a comfortable bed in a dimly lit room, with a temperature of 21–24°C. Pneumatic probes were placed on each index finger and a blood pressure cuff on one arm. With the arms at rest on comfortable arm supports, probes were inflated to a subdiastolic pressure to avoid distal venous pooling, thereby inhibiting the veno-arteriolar vasoconstriction reflex (31). The recording was initiated after 25 min of rest. After 5 min baseline recording, the blood pressure cuff was inflated to 60 mmHg above systolic blood pressure and no less than 200 mmHg. Occlusion was confirmed by visual confirmation of complete attenuation of the PAT signal from the test arm. After 5 min occlusion, the cuff was deflated and the recording continued for 5 min during the reactive hyperemia phase. Recordings from the non-occluded arm served as an internal control correcting for systemic changes in vascular tone.

### Calculation of EF

Endothelial function and arterial stiffness were calculated using the EndoPAT software package version 3.4.4. EF was given as the reactive hyperemia index (RHI), the natural logarithm of RHI (lnRHI), and Framingham RHI (fRHI). Arterial stiffness as the augmentation index (AI) and AI standardized to a pulse of 75/min (AI@75).

Reactive hyperemia index was calculated as the index of signal amplitude pre-to-post occlusion in the occluded arm, divided by the same ratio in the control arm. Calculation of fRHI was done according to Hamburg et al (32). Determination of AI is based on computerized averaging and analysis of multiple pulse waveforms obtained during the baseline measurement on the occluded arm. AI@75 is corrected for differences in pulse by normalization to a heart rate of 75 beats/min.

Reactive hyperemia index, lnRHI, fRHI, AI, and AI@75 data were tested for normality by Shapiro–Wilks test and visual inspection of histograms and Q–Q plots. Differences between mean values were assessed by paired *t*-test. Correlation of same-day and day-to-day measurements was calculated as the Pearson’s product-moment correlation coefficient (Pearson’s *r*) and intraclass correlation coefficients (ICCs). In accordance with Landis and Koch, we interpreted ICC values as follows: >0.80 was almost perfect, 0.61–0.80 was substantial, 0.41–0.60 was moderate, 0.21–0.40 was fair, 0.20–0.00 was slight, and <0.00 was poor (33). The correlation of measurements is presented as Bland–Altman plots. All statistical calculations were performed using Rstudio (Rstudio, Boston, MA, USA) applying standard packages and packages “dplyr,” “BlandAltmanLeh,” “Stats,” and “ICC.”

## Results

We included two different populations that were analyzed separately: (1) healthy individuals (*n* = 20, men 50%, mean age 35.85 ± 3.47 years) and (2) stroke patients (*n* = 22, men 52%, mean age 66.38 ± 2.85 years). Population characteristics are shown in Table 1. Only intraindividual assessment within each group was performed, as difference in response between healthy subjects and stroke patients is already known to exist and not the scope of this paper.

**Table 1:** **Demographic data**.

	Stroke	Healthy
Age (years)	67.27 ± 1.63	35.9 ± 3.47
Sex (male)	0.55	0.50
BMI	25.25 ± 0.49	22.73 ± 0.41
DBP	82.23 ± 1.49	70.82 ± 1.07
SBP	142.72 ± 2.61	118.78 ± 2.02
NIHSS	4.09 ± 0.53	0.00 ± 0.00
Reactive hyperemia index	2.24 ± 0.09	1.93 ± 0.06
Logarithm of RHI	0.76 ± 0.04	0.63 ± 0.03
Framingham RHI	0.57 ± 0.05	0.41 ± 0.05
Augmentation index	27.91 ± 2.75	−3.37 ± 1.76
AI standardized to a pulse of 75/min	21.86 ± 2.34	−12.69 ± 1.74

*All results are given as mean ± SEM*.

All data sets were accepted as suitable for parametric testing. However, it should be noted that according to the manufacturer of the EndoPAT device, RHI data represent a skewed distribution.

### Stroke Patients

We found no significant difference between same-day or day-to-day measurements (*p* = 0.96 and 0.76, respectively). Correlation coefficients showed moderate to substantial reliability of day-to-day measurements with ICC values ranging from 0.56 to 0.72 for EF measurements. Data for same-day measurements showed more varied results with ICC values from 0.25 to 0.64. Repeated measurements of fRHI and lnRHI were more reliable than RHI both on same-day and day-to-day measurements (Table 2). Measurements of arterial stiffness (AI@75) showed almost perfect agreement on same-day (ICC = 0.94) and day-to-day (ICC = 0.91) measurements.

**Table 2:** **Reliability of repeated testing in stroke patients**.

	Mean difference ± SD	*p*-Value	Pearson’s *r*	Intraclass correlation coefficient (95% CI)
**Same-day**				
Reactive hyperemia index (RHI)	0.54 ± 0.14	0.68	0.27	0.25 (−0.18; 0.60)
Logarithm of RHI (lnRHI)	0.22 ± 0.05	0.89	0.41	0.40 (0.00; 0.69)
Framingham RHI (fRHI)	0.27 ± 0.06	0.97	0.64	0.64 (0.32; 0.83)
Augmentation index (AI)	4.83 ± 0.92	0.65	0.96	0.96 (0.91; 0.98)
AI standardized to a pulse of 75/min (AI@75)	5.16 ± 0.88	0.93	0.94	0.94 (0.87; 0.98)
**Day-to-day**				
RHI	0.47 ± 0.58	0.74	0.55	0.56 (0.20; 0.79)
lnRHI	0.19 ± 0.18	0.94	0.60	0.62 (0.29; 0.82)
fRHI	0.26 ± 0.23	0.64	0.72	0.72 (0.44; 0.87)
AI	5.51 ± 4.09	0.51	0.95	0.95 (0.89; 0.98)
AI@75	6.70 ± 3.87	0.15	0.92	0.92 (0.82; 0.97)

**Figure 1:**
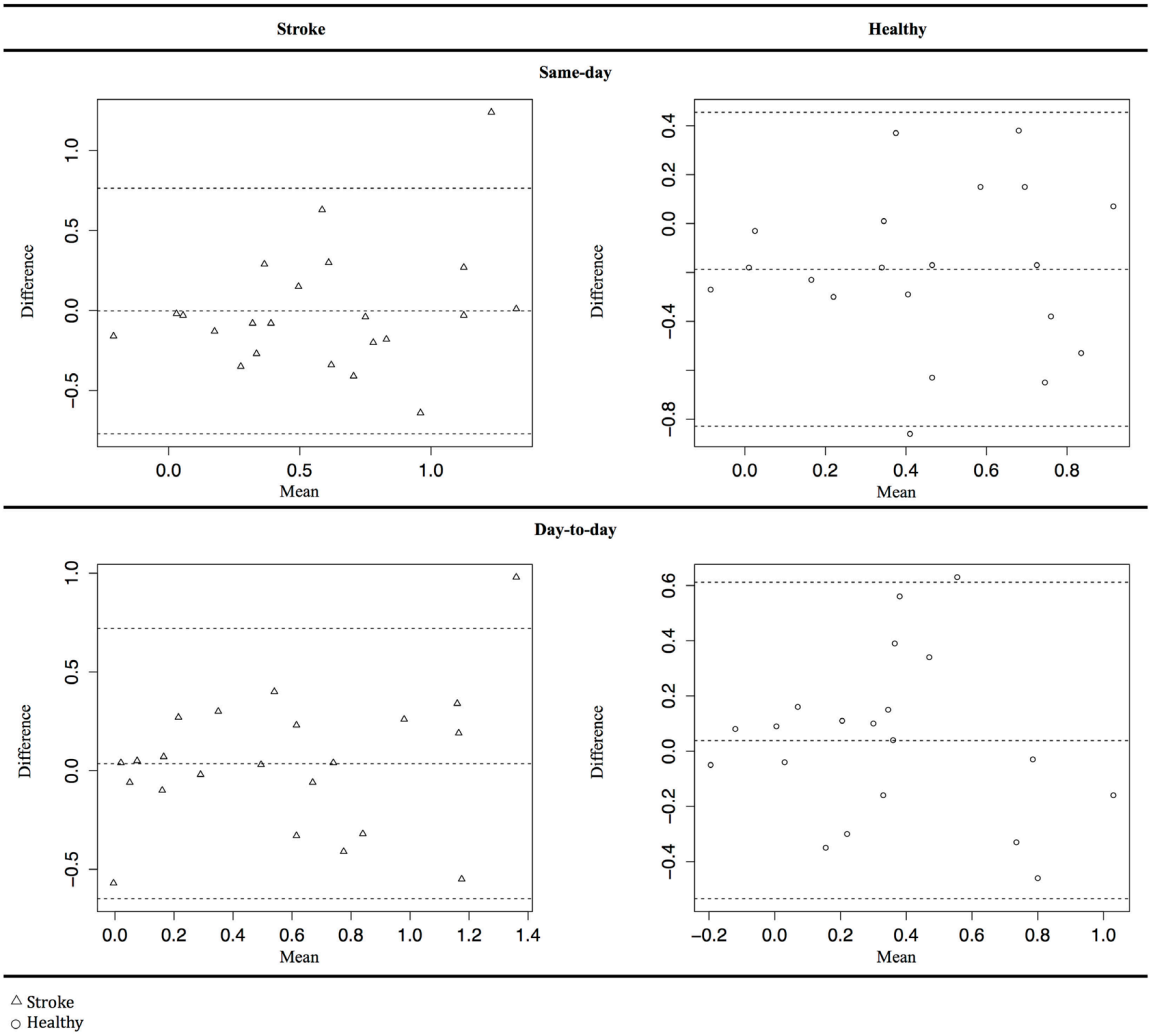
**Bland–Altman plot for Framingham reactive hyperemia index data**. Δ Stroke patients, ○ healthy subjects.

We found no evidence of systematic errors in Bland–Altman plots (Figure 1). However, the plots show one distinct outlier. We analyzed the data for this specific patient and found no errors leading to exclusion of the patient’s data.

### Healthy Subjects

For day-to-day measurements, there was no significant difference between mean values (*p* = 0.82). For same-day measurements, the difference in mean values was close to significant (*p* = 0.05). Correlation coefficients showed moderate to substantial reliability of day-to-day measurements with ICC values ranging from 0.51to 0.67. Same-day measurements showed fair to moderate reliability with ICC values ranging from 0.28 to 0.51 (Table 3). Reliability of arterial stiffness measurements (AI@75) showed substantial agreement on same-day (ICC = 0.76) and an almost perfect agreement on day-to-day measurement (ICC = 0.82). The Bland–Altman plots (Figure 1) show no evidence of systematic error.

**Table 3:** **Reliability of repeated testing in healthy subjects**.

	Mean difference ± SD	*p*-Value	Pearson’s *r*	Intraclass correlation coefficient (95% CI)
**Same-day**				
Reactive hyperemia index (RHI)	0.46 ± 0.28	0.09	0.28	0.23 (−0.22; 0.60)
Logarithm of RHI (lnRHI)	0.23 ± 0.13	0.05	0.37	0.29 (−0.16; 0.64)
Framingham RHI (fRHI)	0.30 ± 0.22	0.02	0.51	0.42 (−0.01; 0.72)
Augmentation index (AI)	6.86 ± 6.84	0.90	0.81	0.79 (0.56; 0.91)
AI standardized to a pulse of 75/min (AI@75)	7.74 ± 6.97	0.34	0.78	0.76 (0.49; 0.90)
**Day-to-day**				
RHI	0.39 ± 0.30	0.94	0.49	0.51 (0.10; 0.77)
lnRHI	0.20 ± 0.15	0.82	0.51	0.52 (0.13; 0.78)
fRHI	0.23 ± 0.18	0.56	0.66	0.67 (0.33; 0.85)
AI	5.18 ± 3.62	0.84	0.87	0.87 (0.70; 0.94)
AI@75	6.16 ± 3.83	0.22	0.84	0.82 (0.61; 0.93)

## Discussion

This study provides interesting data on the reliability of EF testing in patients with acute stroke by measuring the digital vascular response to reactive hyperemia. We found moderate to substantial correlation on day-to-day EF measurements in both healthy subjects and stroke patients.

We found worse correlations of same-day repeated measurements in both groups. Same-day measurements in healthy subjects also showed trends toward a difference in EF.

The digital vascular response is calculated from the change in pulse wave amplitude caused by vasodilation in response to reactive hyperemia. If an ample amount of time between measurements is not applied for the vessels to regain vascular steady state, a carryover effect may influence the subsequent measurement. This could explain the poor correlation of same-day measurements in both groups.

We found that in healthy subjects EF measurements showed a worse correlation between test and retest compared with the stroke patients. Such correlation could reflect a larger heterogeneity of the healthy subjects than of the stroke patients; the mean age was significantly lower in healthy subjects, with more widespread age distribution within the group compared with the stroke patients. However, due to the modest sample size of this study we cannot discard that the difference may be caused by random variation.

Previous studies have assessed reliability of EF measurements using EndoPAT, in healthy individuals and patients with CAD (Table 4). Similar to our findings, these studies found substantial reliability of EF measurements performed on two separate days reporting ICC values between 0.56 and 0.83 (14, 24–27). Only two studies investigated the reliability of same-day measurements although with diverging results. One reported good correlation of repeated measurements after 30 min (ICC 0.68) (14), while the other study indicated a possible carryover effect of repeated measurements after 30 min (28).

**Table 4:** **Comparison with studies reporting test–retest reliability**.

Reference	Number of patients (M/F)	Patients	Mean age	Interval between measurements	Measured value	Pearson’s *r*	ICC
Hansen et al. (current study)	20 (10/10)	Healthy	35.9 ± 3.47^a^	1.5 h	fRHI	0.51	0.42
					AI@75	0.78	0.76
				24 h	fRHI	0.66	0.67
					AI@75	0.84	0.82
Hansen et al. (current study)	22 (12/10)	Acute stroke patients	67.27 ± 1.63^a^	1.5 h	fRHI	0.64	0.64
					AI@75	0.94	0.94
				24 h	fRHI	0.72	0.72
					AI@75	0.92	0.92
Tomfohr et al. (24)	12 (11/1)	Healthy	26.8	1–7 days	–	0.76	0.73
Reisner et al. (25)	113 (73/40)	Healthy	40 ± 12.6^b^	1 day	fRHI	0.56	0.56
					AI	0.84	0.84
McCrea et al. (26)	20 (14/6)	Disease free overweight	41.2 ± 2.4^a^	Mean 19.5 ± 6.2^a^ days	RHI	0.68	0.74
					fRHI	0.77	0.77
					AI	0.89	0.83
					AI@75	0.88	0.81
Selamet Tierney et al. (27)	30 (17/13)	Healthy	17.3 (13.3–19.7)^c^	>7 days	RHI	–	0.78
					fRHI	–	0.83
Onkelinx et al. (14)	18 (18/0)	CAD	68.3 ± 7.8^b^	30 min	RHI	–	0.68
				2 days	RHI	–	0.73

*M, male; F, female; ICC, intraclass correlation coefficient; fRHI, Framingham reactive hyperemia index; AI@75, AI normalized to pulse 75/min; AI, augmentation index; RHI, reactive hyperemia index; CAD, coronary artery disease*.

*^a^Mean ± SEM*.

*^b^Mean ± SD*.

*^c^Mean (range)*.

The study in CAD patients (14) is of particular interest in relation to our study, as these patients have known vascular disease but show good correlation of both day-to-day and same-day measurements. However, the group of patients in this study would be assumed to have a steady vascular function because they had no cardiovascular incident 9 months prior to testing. No other studies seem to have investigated reliability of measurements in patients with acute vascular disease.

Endothelial cell activation and vascular variability have gained increasing interest in stroke research (29, 30). Thus, investigating the reliability of EF testing in the acute phase poststroke is important for future studies in this area. Compared with other methods used for assessing EF, the use of the digital PAT has the advantage of being almost operator independent, with standardized measurements and calculations. In addition, the technique is simple and requires minimal training of the operator, thus easily accessible for large-scale investigations of risk factors associated with endothelial dysfunction and for testing the effect of vasoactive agents.

Two large community-based cohorts have investigated the association between RHI score and known cardiovascular risk factors (5, 21). They found poor EF to be associated with metabolic risk factors including higher BMI, higher cholesterol, diabetes, smoking, and female sex (5, 21). Assuming that digital EF score correlates with metabolic risk factors, which are key risk factors for both large and small vessel disease (34), this method could be used to assess the risk of such diseases.

The usability of digital EF measurements for testing vascular effects of new treatments, assessing stroke prognosis, and individualizing stroke prevention is still questionable, however, promising. It is mandatory to apply this technique on larger cohorts of stroke patients to assess the relation between endothelial dysfunction and risk factors for stroke. In addition, studies investigating the association of this EF score with stroke subtypes are needed as this has only been investigated by studies with small sample sizes (23, 35).

### Strengths and Limitations

Strict standardized conditions were applied during testing to minimize confounders (36). All stroke patients were enrolled within the first 3 days, and tests were completed within 5 days of stroke onset, to lower the effect of difference in vascular instability during the acute phase poststroke.

Since a previous study showed good reliability in patients with cardiovascular disease (14), we did not correct for known cardiovascular disease. Also, none of the included patients had a history of recent cardiovascular events prior to the stroke. We did, however, exclude patients with paroxysmal atrial fibrillation, as studies have shown atrial fibrillation to be associated with improved EF upon re-establishment of sinus rhythm (37, 38).

Our study was designed to assess the reliability of repeated measurements preformed on the same patient/subject. Thus, fallacies caused by differences between the healthy subjects and patients as well as within-group differences, e.g., use of vasoactive medication, should not affect the main result significantly but can be of interest for generating hypotheses for future research.

We cannot assess, based on the current data, differences in EF in relation to burden of stroke severity. All stroke patients included in this study had minor to moderate stroke with a mean NIHSS of 4.09 (range 0–8). It is possible that patients with more severe strokes have increased vascular affection due to greater endothelial cell activation and increased vascular instability. The reliability in patients with severe stroke may thus be different from our results warranting further research into this area.

## Conclusion

Endothelial function measurements in particular fRHI using PAT technology are reliable in patients with acute stroke and in healthy subjects using day-to-day measurements. Measurements of arterial stiffness showed substantial to almost perfect correlations independent of the time between measurements in both groups. A carryover effect of repeated same-day measurements cannot be excluded, if these are not separated by an ample amount of time. Based on our results, we recommend EF measurements to be performed at the same time of the day on two separate days or with ample time between on same-day to achieve reliable data.

## Ethics Statement

The ethics committee in the capital region of Denmark and the Danish Data Protection Agency approved this study.

## Author Contributions

AH, JB, SH-Y, WK, and CK contributed to the study design, data acquisition, and data analysis. AH and CK drafted the manuscript. All authors interpreted the results, revised the manuscript, and approved the final version.

## Conflict of Interest Statement

The authors declare that the research was conducted in the absence of any commercial or financial relationships that could be construed as a potential conflict of interest.
